# Anterior Lamina Cribrosa Surface Depth in Open-Angle Glaucoma: Relationship with the Position of the Central Retinal Vessel Trunk

**DOI:** 10.1371/journal.pone.0158443

**Published:** 2016-06-29

**Authors:** Baek-Lok Oh, Eun Ji Lee, Hyunjoong Kim, Michaël J. A. Girard, Jean Martial Mari, Tae-Woo Kim

**Affiliations:** 1 Department of Ophthalmology, Seoul National University College of Medicine, Seoul National University Bundang Hospital, Seongnam, Korea; 2 Department of Applied Statistics, Yonsei University, Seoul, Korea; 3 Department of Biomedical Engineering, National University of Singapore, Singapore, Singapore; 4 Singapore Eye Research Institute, Singapore National Eye Centre, Singapore, Singapore; 5 GePaSud, Université de la Polynésie Française, Faaa, French Polynesia; Centre for Eye Research Australia, AUSTRALIA

## Abstract

**Purpose:**

To determine the factors influencing the anterior lamina cribrosa (LC) surface depth (LCD) in patients with open-angle glaucoma (OAG), focusing on the association between LCD and the position of the central retinal vessel trunk (CRVT) at the anterior LC surface.

**Methods:**

Optic nerve heads of 205 OAG eyes were scanned using swept-source optical coherence tomography (SS-OCT). After processing the images using adaptive compensation, the LCD was determined from 11 horizontal B-scan images that divided the optic disc vertically into 12 equal parts. Eyes were divided into two groups (central or peripheral) according to where the CRVT exits from the anterior LC surface. The influence of CRVT position on LCD was evaluated, taking into account age, gender, untreated intraocular pressure (IOP), IOP at optic-disc scanning, retinal nerve fiber layer (RNFL) thickness, visual-field mean deviation, central corneal thickness, and axial length.

**Results:**

Patients in the peripheral CRVT group were younger and more myopic, and had a larger mean LCD and thinner global RNFL than those in the central CRVT group (all *P*≤0.023). On multivariate analysis, the peripheral CRVT location was significantly associated with a larger LCD (*P* = 0.002), together with the significant association of younger age (*P*<0.001), higher untreated IOP (*P* = 0.010), and thinner RNFL (*P* = 0.003) on the larger LCD.

**Conclusion:**

In OAG, CRVT location was an independent factor influencing the LCD, together with age, untreated IOP, and global RNFL thickness. The data indicate that the CRVT may contribute to the resistance of the LC against deformation. A longitudinal prospective observation is required to clarify this relationship.

## Introduction

The lamina cribrosa (LC) is the primary site of glaucomatous optic nerve damage. Histologic studies have demonstrated that compression and posterior displacement of the LC underlies glaucomatous cupping [1, 2]. The deformation of the LC may cause kinking and pinching of the axons passing through the laminar pores, promoting blockade of the axoplasmic flow [3–5]. In addition, LC deformation may compress the laminar capillaries, thereby causing ischemic insult to the axons [1, 6]. Experimental studies have shown that displacement of the LC precedes early surface-detected structural damage and retinal nerve fiber layer (RNFL) loss [7–10]. This finding further suggests that LC deformation is the primary event underlying axonal damage in glaucoma.

Elevation of the anterior LC surface at the center of the optic nerve, termed the central horizontal ridge of the LC, has recently been demonstrated using enhanced depth imaging spectral-domain optical coherence tomography (EDI SD-OCT) [11]. It has been postulated that the central LC ridge might be associated with the connective tissue surrounding the central retinal vessel trunk (CRVT) [11, 12], and that it might act as a structural support against LC deformation [11]. This assumption is supported by several previous investigations that revealed correlations between an eccentrically located CRVT, and the pattern of glaucomatous rim loss [13], or the location of parapapillary atrophy in open-angle glaucoma (OAG) [14]. Huang et al [15]. recently showed that the residual visual-field pattern in advanced glaucoma was associated with the CRVT position, such that the central visual field was less likely to be affected in eyes with a CRVT that is located centrally within the optic disc. Based on these previous findings, it can be hypothesized that if the CRVT could act as a supporting structure for the LC against deformation, an LC with a CRVT near its center would be more resistant to displacement by mechanical stress than an LC with a CRVT located at the periphery.

The precise position of the CRVT within the LC should be determined by taking into account the entire LC surface. However, limitations regarding the currently available methods of examining the entire LC in patients have resulted in the location of the CRVT thus far being determined based only on the clinical optic-disc margin. However, the clinical optic-disc margin is not a consistent structure and its reported location varies among observers. Although EDI SD-OCT enables visualization of the detailed structure of the LC, visualization of the entire LC remains problematic because the vascular shadow or Bruch’s membrane lying over the LC often obscures the LC signal, particularly near its insertion [16]. On the other hand, swept-source OCT (SS-OCT) uses a laser operating at a longer wavelength compared with SD-OCT. The consequently deeper tissue penetration achieved with SS-OCT may enable visualization of the entire LC to the periphery, enabling a more precise localization of the CRVT within the LC.

The purpose of the present study was to determine whether the CRVT can act as a stabilizing structure against LC deformation. To this end, the location of the CRVT within the LC was determined using SS-OCT images of the optic nerve head (ONH), and the influence of CRVT location on the anterior LC surface depth (LCD) was evaluated in OAG patients.

## Materials and Methods

This investigation was based on the Investigating Glaucoma Progression Study (IGPS), which is an ongoing prospective study at the Seoul National University Bundang Hospital Glaucoma Clinic. The study was approved by the Seoul National University Bundang Hospital Institutional Review Board and conformed to the Declaration of Helsinki. Written informed consent to participate was provided by all patients.

### Study Subjects

Patients with OAG who were enrolled in the IGPS between January 2013 and December 2014 received comprehensive ophthalmic examinations that included visual acuity assessment, refraction, Goldmann applanation tonometry, slit-lamp biomicroscopy, gonioscopy, and dilated stereoscopic examination of the ONH. They also submitted to measurement of central corneal thickness (Orbscan II, Bausch & Lomb Surgical, Rochester, NY, USA), axial length (AXL; IOL Master v. 5, Carl-Zeiss Meditec, Dublin, CA, USA), and corneal curvature (KR-1800, Topcon, Tokyo, Japan), disc photography (EOS D60 digital camera, Canon, Utsunomiyashi, Tochigken, Japan), SD-OCT circumpapillary retinal nerve fiber layer (RNFL) thickness measurement (Spectralis OCT, Heidelberg Engineering, Heidelberg, Germany), SS-OCT scanning of the optic disc (DRI-OCT1 Atlantis, Topcon), and standard automated perimetry (24–2 Swedish interactive threshold algorithm; Humphrey Field Analyzer II 750, Carl-Zeiss Meditec).

The inclusion criteria were (1) presence of OAG, (2) a best-corrected visual acuity of 20/40 or better, (3) a spherical equivalent ranging from –8.0 D to +3.0 D and a cylinder correction ranging from –3.0 D to +3.0 D, and (4) good-quality OCT images that allowed clear delineation of the anterior LC surface including the periphery near the LC insertion. OAG was defined as having an open angle on gonioscopy, glaucomatous optic-nerve damage (i.e., the presence of focal thinning of the neuroretinal rim or notching), and associated visual-field defects without ocular disease or conditions that may elevate IOP. A glaucomatous visual-field change was defined as (1) outside the normal limits on the Glaucoma Hemifield Test; (2) three adjacent abnormal points with a <5% probability of being normal, 1 with *P*<1% by pattern deviation; or (3) a pattern standard deviation of <5% if the visual field was otherwise normal, as confirmed on two consecutive tests. The exclusion criteria were (1) eyes with optic-disc tilt (defined by a tilt ratio—the ratio between the longest and shortest diameters of the optic disc—of >1.3) [17], (2) a torted optic disc (defined as a torsion angle—deviation of the long axis of the optic disc from the vertical meridian—of >15°) [18], (3) unreliable visual-field tests (fixation loss rate >20%, or false-positive or false-negative error rate >25%), (4) a history of ocular surgery including glaucoma surgery, other than cataract surgery, or (5) presence of intraocular diseases (e.g., diabetic retinopathy or retinal vein occlusion) or neurologic diseases (e.g., pituitary tumor) that could cause visual-field loss.

Untreated IOP was defined based on the average value of at least two measurements made within 2 weeks before the initiation of IOP-lowering treatment. When patients were being treated with ocular hypotensive medication at the time of the initial visit, the untreated IOP was measured after a 4-week washout period. The scan IOP was defined as the IOP measured on the day of SS-OCT examination. When both eyes of a single patient were eligible, the data for one eye were randomly chosen for data analysis.

### SS-OCT Scanning of the Optic Disc

SS-OCT was performed using the DRI-OCT1 Atlantis system (Topcon) [19–22]. This method of OCT uses a light source of a wavelength-sweeping laser centered at 1050 nm with a repetition rate of 100 kHz, yielding an 8-μm axial resolution in tissue. Images were obtained first using the 6-mm, 12-radial-scan protocol centered on the ONH. Each radial scan was obtained by averaging up to 32 single images. Then, 3-dimensional (3D) raster scan protocol consisting of 512 × 256 A-scans was acquired over a square area of 6 mm × 6 mm covering the entire ONH. The speckle noise was reduced by applying weighed moving average from three consecutive single images. Adjustment was made for the magnification error by entering the AXL and keratometry values before image acquisition.

SS-OCT images that were obtained at least more than 6 months after initiating IOP-lowering treatment were used for the analysis. LCD may be reversed (i.e., forward shifting of the anterior LC surface) after initiating IOP-lowering treatment [23], thus during the LC reversal, the LC measurements could be variable according to the time course. In our experience, if such a reversal occurs, it is mostly complete by 3 months after IOP-lowering therapy [23]. Hence, we thought that the 6-months window represents a good cutoff point for evaluating a relatively ‘stable’ LCD.

### Determination of the Position of CRVT Within the LC

The location of the CRVT within the LC was assessed using the radial-scan images. First, the image containing the CRVT, which manifests as a hyporeflective shadow penetrating the LC [24], was selected from the 12 radial-scan images. An adaptive intensity compensation algorithm was then applied to improve the visibility of the peripheral LC. The compensation algorithm was implemented to compensate for light attenuation, and the contrast enhancement algorithm was implemented to enhance tissue contrast in the OCT images. These algorithms have been shown to significantly improve the visibility of the peripheral LC including the LC insertions [16, 25, 26].

**Fig 1:**
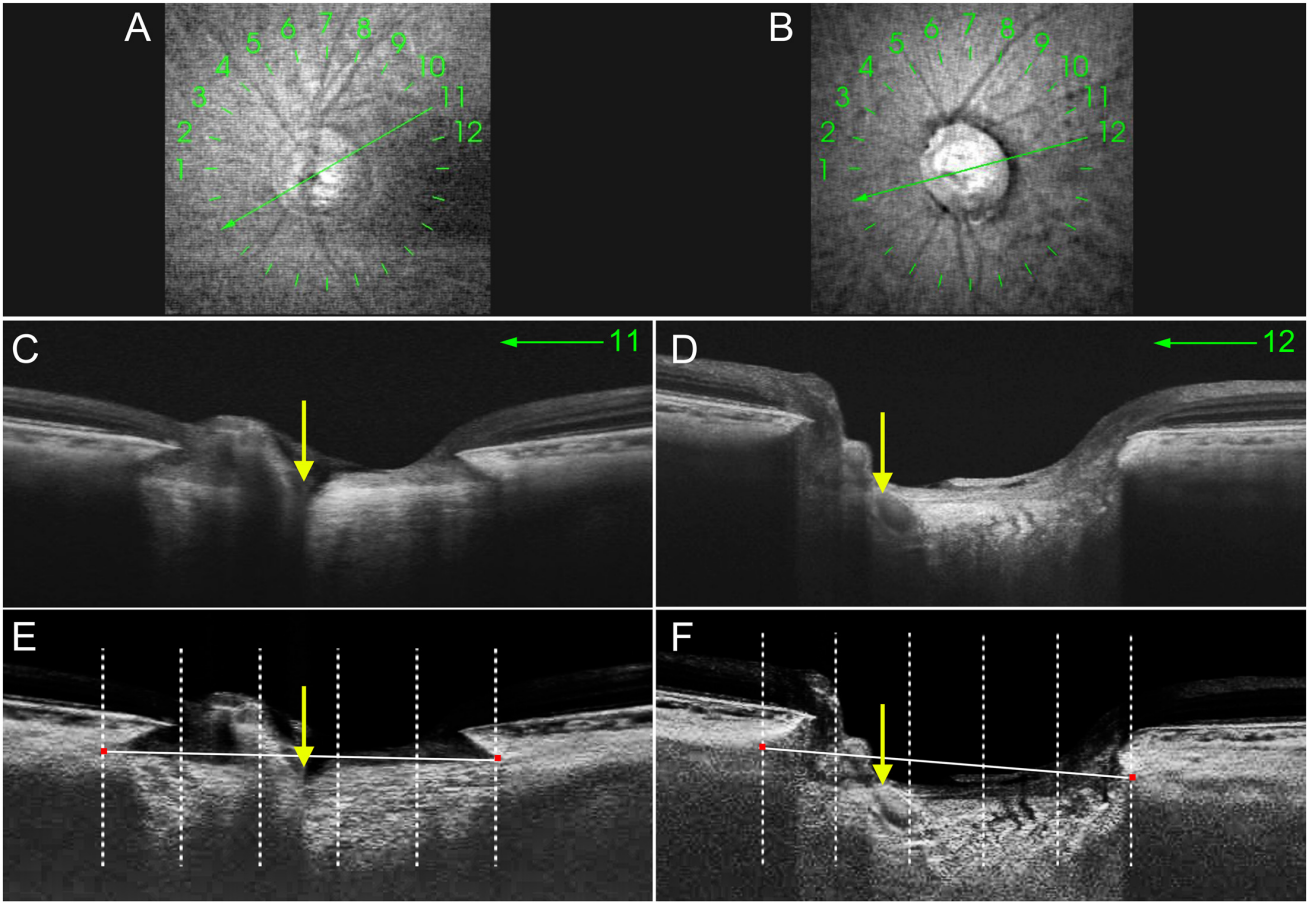
Determination of the position of central retinal vessel trunk (CRVT) within the lamina cribrosa (LC) using swept-source optical coherence tomography (SS-OCT) radial-scan images. **A, B** SS-OCT infrared images of eyes with a central CRVT (**A**) and a peripheral CRVT (**B**). The *green markings with numbers* indicate the location at which the radial images were obtained. Images obtained at locations 11 (**A**) and 12 (**B**) were used to determine the CRVT position for each eye. **C, D** B-scan images containing the CRVT. The CRVT is shown as a hyporeflective shadow penetrating the LC (*yellow arrows*). **E, F** These images are the same as the above B-scan images after adaptive compensation. *Red glyphs* indicate the point of anterior LC insertion and *vertical dashed lines* indicate the locations dividing the anterior LC surface diameter (*white solid lines*) into five equal parts. Determination of the CRVT position was based on the location of the CRVT exit at the level of the anterior LC surface. (**E**) The CRVT exit in this eye is located within the central one-fifth of the anterior LC surface diameter (*yellow arrow*), and thus this eye was categorized into the central group. (**F**) The CRVT of this eye is outside the central one-fifth of the anterior LC surface diameter (*yellow arrow*), and thus this eye was assigned to the peripheral group. Note that the anterior LC surface depth is smaller in the eye with a central CRVT (**E**) than in that with a peripheral CRVT (**F**).

The processed images were then analyzed by image-processing software (Image J, version 1.31, Wayne Rasband, Research Services Branch, National Institute of Mental Health, Bethesda, MA, USA) to determine the position of the CRVT based on the location of the CRVT exit at the level of the anterior LC surface. When both the central retinal artery and vein were located peripheral to the central one-fifth of the anterior LC surface diameter, the eye was categorized as having a peripheral CRVT, while at least one of them were located within the central one-fifth of the anterior LC surface diameter, the eye was categorized as having a central CRVT (Fig 1). When the CRVT met the demarcation line indicating the central one-fifth of the anterior LC surface diameter, the CRVT location was determined based on the side where more than half of the CRVT area belonged to: When more than half of the diameters of central retinal artery or vein was deviated peripherally from the demarcation line, the eye was categorized as peripheral CRVT group. The CRVT position was evaluated independently by two clinicians (B.L.O. and E.J.L.) who were masked to the patients’ clinical information, including their LC measurements. The final decision was determined by consensus agreement between the two observers.

### Measurement of the LCD

**Fig 2:**
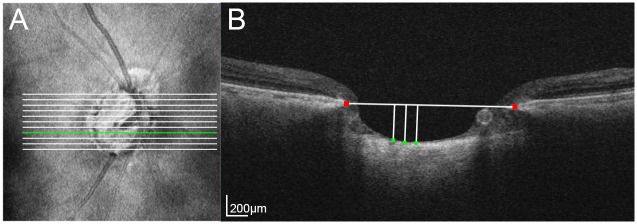
Measurement of the anterior lamina cribrosa (LC) surface depth (LCD). **A.** Infrared image indicating the locations where the 11 B-scan images were selected for the LCD measurements (horizontal lines). **B.** B-scan image at the location corresponding to the green horizontal line in panel A. The LCD was determined by measuring the distance from Bruch’s membrane opening plane to the level of the anterior LC surface. A reference line connecting the two termination points of Bruch’s membrane (red glyphs) was drawn in each B-scan, and the distance from that reference line to the level of the anterior border of the LC was measured at three points (green glyphs). The average of the three values was defined as the LCD in the selected B-scan.

The LCD was measured on the 3D horizontal raster scan images, as described elsewhere [27, 28], using the manual caliper tool incorporated into the DRI-OCT1 Atlantis system. The LCD was determined by measuring the distance from Bruch’s membrane opening plane to the level of the anterior LC surface. To this end, a reference line connecting the two termination points of Bruch’s membrane was drawn in each B-scan, and the distance from that reference line to the level of the anterior border of the LC was measured at three points: the maximally depressed point and two additional points (100 and 200 μm from the maximally depressed point in the temporal direction). Only temporally adjacent points were selected because the maximally depressed point was often close to the CRVT, the shadow of which obscured the LC. The average of the three values was defined as the LCD of the selected B-scan (Fig 2).

The mean LCD was determined as the average of the LCD measurements from 11 B-scan images that divided the optic-disc diameter of each eye vertically into 12 equal parts, from the 3D image data set (Fig 2). The average of the measurements obtained from the three central-most B-scans was defined as the central LCD.

### Statistical Analysis

The interobserver agreement for categorizing the CRVT position was evaluated using Cohen’s kappa statistic. The interobserver reproducibility of the LCD measurement was determined by calculating the intraclass correlation coefficient (ICC) and its confidence interval (95%CI). The independent-samples *t*-test and chi-square test were used to compare the data between the central and peripheral groups. A general linear model was used to determine the factors associated with the LCD. Fisher’s z-test was used to compare the correlations of the LCD with several parameters between groups. Statistical analyses were performed using the Statistical Package for the Social Sciences version 20.0 (SPSS, Chicago, IL, USA). The cutoff for statistical significance was set at *P*<0.05, and except where specified otherwise, the data are expressed as mean ± standard deviation values.

## Results

Of the 227 eyes of 227 OAG patients that were initially included, 22 eyes were excluded because of poor OCT image quality, whereby the LC insertion could not be identified clearly even after compensation. The anterior LC surface and the peripheral LC were clearly discernible in all 205 of these included eyes.

The patients were aged 54±15 years (range, 16–88 years) and comprised 84 women and 121 men. The refractive error (spherical equivalent), untreated IOP, scan IOP, and visual-field mean deviation of the entire cohort were –1.92±3.08 D (range, –8.00 to +3.00 D), 17.8±7.5 mmHg (range, 7–52 mmHg), 12.0±2.6 (range, 5–22 mmHg), and –8.14±8.29 dB (range, –33.52 to +1.58 dB), respectively.

The interobserver agreement for determining the location of the CRVT was excellent (Cohen’s kappa = 0.95). The interobserver ICC for measurement of the LCD was 0.987 (95%CI = 0.983–0.990).

Of the 205 included eyes, 147 (71.7%) were categorized as having a central CRVT and 58 eyes (28.3%) as having a peripheral CRVT. Patients in the peripheral group were younger (age 49±15 years *vs*. 56±15 years, *P* = 0.002), more myopic (–3.64±3.10 D *vs*. –1.23±2.80 D, *P*<0.001), and had a greater AXL (25.65±1.79 mm *vs*. 24.38±1.24 mm, *P*<0.001) than those in the central group. The mean and central LCDs were significantly greater in the peripheral group than in the central group (585.81±174.85 μm *vs*. 500.62±130.90 μm, *P*<0.001, and 591.09±185.21 μm *vs*. 488.52±145.54 μm, *P*<0.001, respectively). The global RNFL was thinner in the peripheral group than in the central group (67.29±16.95 *vs*. 73.50±17.58 μm, *P* = 0.023). There were no significant differences between the two groups with respect to gender, laterality, untreated IOP, scan IOP, visual-field mean deviation and pattern standard deviation, and central corneal thickness (all *P*>0.05; Table 1).

**Table 1:** Demographics and baseline characteristics.

	RVT central group (n = 147)	RVT peripheral group (n = 58)	*P-value*
Gender: male	85 (57.8%)	36 (62.1%)	0.638*
Laterality: right	73 (49.7%)	29 (50.0%)	1.000*
Mean LCD (μm)	500.62 ± 130.90	585.81 ± 174.85	**<0.001**^†^
Central LCD (μm)	488.52 ± 144.54	591.09 ± 185.21	**<0.001**^†^
Age (yrs)	56 ± 15	49 ± 15	**0.002**^†^
Untreated IOP (mmHg)	17.7 ± 7.2	17.8 ± 8.2	0.929^†^
Scan IOP (mmHg)	12.1 ± 2.8	11.9 ± 2.2	0.626^†^
VF MD (dB)	-7.98 ±8.40	-8.55 ± 8.04	0.662^†^
VF PSD (dB)	6.40± 4.60	6.49 ± 4.15	0.895^†^
CCT (μm)	558 ± 37	558 ± 33	0.919^†^
AXL (mm)	24.38 ± 1.24	25.65 ± 1.79	**<0.001**^†^
Refractive error (D)	-1.23 ± 2.80	-3.64±3.10	**<0.001**^†^
Global RNFL thickness (μm)	73.50 ± 17.58	67.29 ± 16.95	**0.023**^†^

RVT = retinal vessel trunk; LCD = anterior lamina cribrosa surface depth; IOP = intraocular pressure; VF = visual field; MD = mean deviation; PSD = pattern standard deviation; CCT = central corneal thickness; AXL = axial length; D = diopter; RNFL = retinal nerve fiber layer.

*The comparison was performed using Chi-square test

^†^The comparison was performed using independent t-test

The factors influencing the mean LCD were determined using linear regression analysis (Table 2). Univariate analysis revealed that younger age, male gender, a higher untreated IOP, worse visual-field mean deviation, thinner RNFL, and a peripheral CRVT were significantly associated with a greater mean LCD (all *P*≤0.003; Table 2). Multivariate analysis using a general linear model confirmed the significance of the association between mean LCD and younger age (*P*<0.001), higher untreated IOP (*P* = 0.010), thinner RNFL (*P* = 0.003), and peripheral CRVT position (*P* = 0.002).

**Table 2:** Factors influencing the mean LC depth.

	*Univariate*			*Multivariate*		
	Beta 1	95% CI	*P*-value	Beta 1	95% CI	*P*-value
Age, *per 1 year older*	-3.43	(-4.75,-2.15)	**<0.001**	-2.97	(-4.32,-1.63)	**<0.001**
Female gender	-61.97	(-104.75,-20.89)	**0.003**	-34.58	(-109.38,26.08)	0.053
RVT, *peripheral location*	85.19	(40.60,130.03)	**<0.001**	64.33	(-3.71,129.15)	**0.002**
Untreated IOP, *per 1 mmHg higher*	8.40	(5.81,10.91)	**<0.001**	3.55	(0.73,6.15)	**0.010**
Scan IOP, *per 1 mmHg higher*	-5.15	-14.58, 1.78	0.194			
VF MD, *per 1dB worse*	6.02	(3.66,8.42)	**<0.001**	0.96	(-2.54,4.57)	0.589
CCT, *per 1 μm thicker*	-0.42	(-1.02,0.14)	0.152			
AXL, per 1 mm longer	13.62	(-0.10,27.77)	0.053	-13.28	(-27.16,0.80)	0.060
Global RNFL thickness, *per 1 μm thinner*	3.95	(2.96,5.08)	**<0.001**	2.57	(0.91,4.39)	**0.003**

LC = lamina cribrosa; RVT = retinal vessel trunk; IOP = intraocular pressure; VF = visual field; MD = mean deviation; CCT = central corneal thickness; AXL = axial length; RNFL = retinal nerve fiber layer.

**Fig 3:**
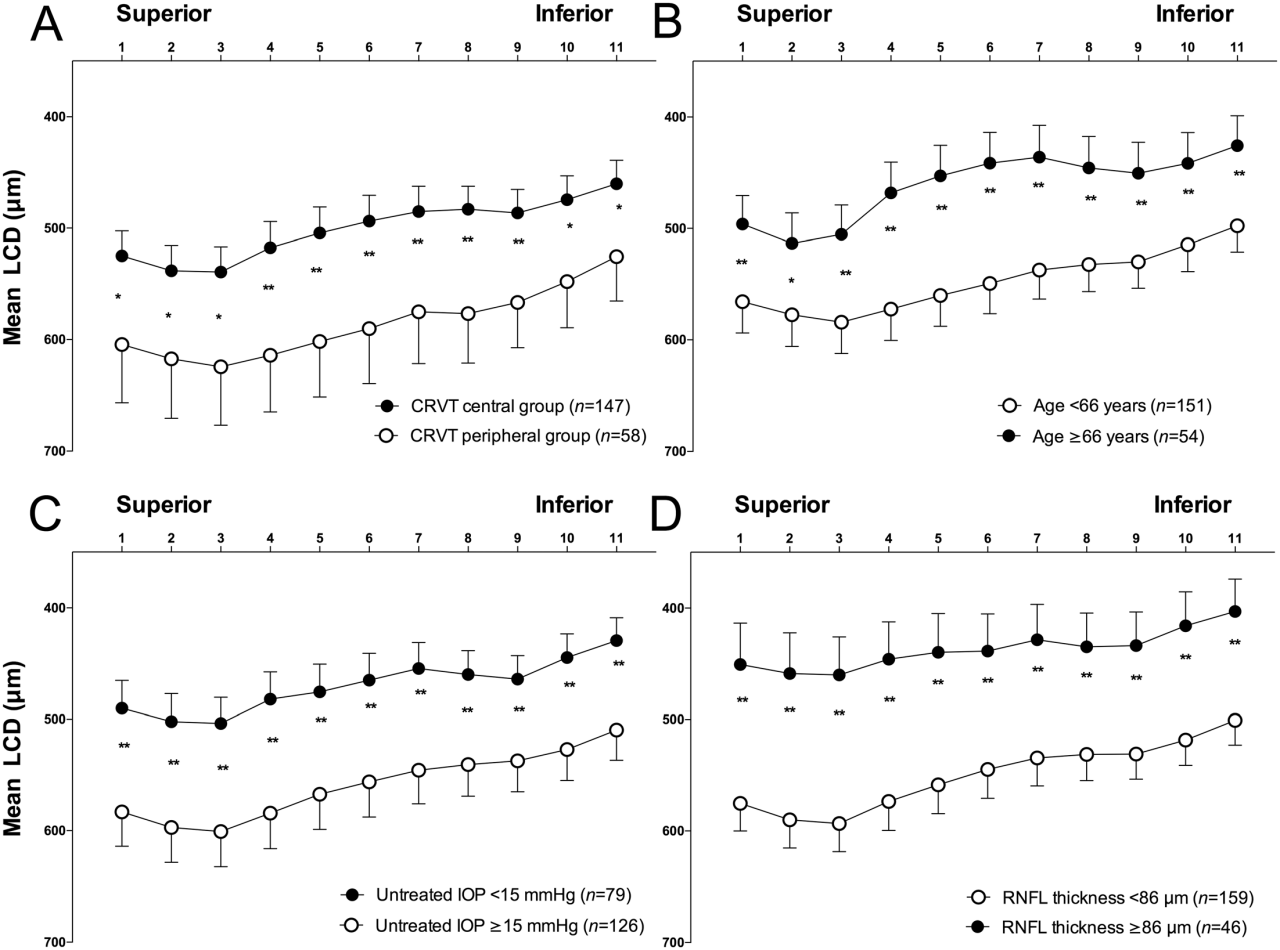
Anterior lamina cribrosa surface depth (LCD) profiles in the 11 horizontal equidistant B-scan images (scans 1–11 are from superior to inferior) according to central retinal vessel trunk (CRVT) location (A), age (B), untreated intraocular pressure (IOP; C), and global retinal nerve fiber layer (RNFL) thickness (D). The cutoff values for continuous variables (age, untreated IOP, and RNFL thickness) for dividing the eyes into two subgroups were obtained by the regression-tree method using GUIDE Classification and Regression Trees and Forests software. Note that overall the anterior lamina cribrosa surface is W-shaped with a central elevation, or has a sloped or focally concave configuration without a central elevation. Error bars represent the 95% confidence interval. *Significantly different at *P*≤0.01; **significantly different at *P*≤0.001.

Fig 3 shows the LCD profiles of 11 equidistant horizontal B-scan images (from superior to inferior) in subgroups divided according to the location of the CRVT, age, untreated IOP, and global RNFL thickness. The cutoff values for continuous variables (age, untreated IOP, and RNFL thickness) were obtained by the regression tree method using GUIDE Classification and Regression Trees and Forests software (version 14.0, available at http://www.stat.wisc.edu/~loh/guide.html) [29]. This analysis provided the cutoff values that best explained the differences in LCD between the two groups. The age of 66 years, an untreated IOP of 15 mmHg, and a global RNFL thickness of 86μm were revealed to be the best cutoff values in this study cohort.

The overall LC contour was tilted superiorly, with varying degrees of central elevation, assuming a W-shape or a focally concave with slope configuration, as described previously [27, 28]. The LCD was significantly larger at all 11 locations in eyes with a peripheral CRVT (Fig 3A), a younger age (Fig 3B), a higher untreated IOP (Fig 3C), and a thinner global RNFL thickness (Fig 3D). Patients in subgroups with a larger LCD exhibited a less distinct central LC elevation (Fig 3).

**Fig 4:**
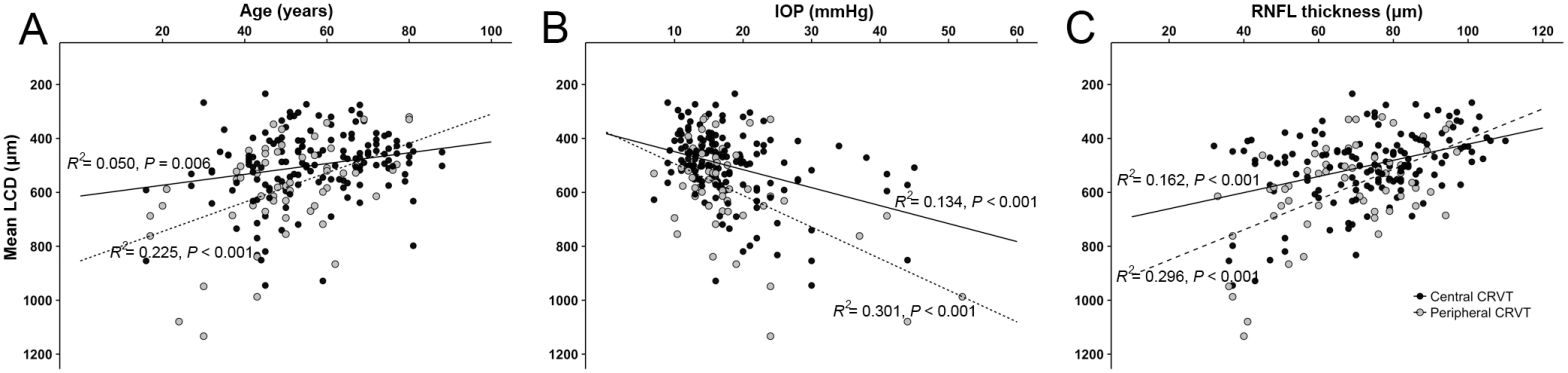
Correlation between the mean anterior lamina cribrosa surface depth (LCD) and age (A), untreated intraocular pressure (IOP; B), and retinal nerve fiber layer (RNFL) thickness (C), according to the location of the central retinal vessel trunk (CRVT). Note that the correlations between age and mean LCD (**A**), and between untreated IOP and mean LCD (**B**) were more prominent in the peripheral CRVT group than in the central CRVT group.

Fig 4 illustrates the relationship between the mean LCD and age (Fig 4A), untreated IOP (Fig 4B), and RNFL thickness (Fig 4C) in the central and peripheral CRVT groups. The correlation between age and mean LCD, between untreated IOP and mean LCD, and between RNFL thickness and mean LCD tended to be stronger in the peripheral CRVT group than in the central CRVT group, but the difference did not reach statistical significance (*P* = 0.070, 0.142 and 0.248, respectively).

## Discussion

Factors influencing LCD were investigated in this study, with a focus on the influence of CRVT location so as to determine its role in the stability of LC. The results revealed an association between a greater LCD and a peripherally located CRVT, younger age, higher untreated IOP, and thinner global RNFL.

It has long been suggested that the location of CRVT within the ONH is associated with the regional vulnerability of the ONH to glaucomatous damage [13, 14]. Jonas et al [13]. showed that a greater distance between the CRVT and the neuroretinal rim was correlated with a thinner neuroretinal rim and a greater degree of parapapillary atrophy [14]. Huang et al [15]. later demonstrated that eyes with a concentric CRVT had a higher chance of maintaining central vision. From a biomechanical perspective, it has been postulated that the CRVT within the ONH may act as a stabilizing element against the glaucomatous changes that occur in the LC [13–15]. On the other hand, a decreased vascular supply from the CRVT to the area distant from the CRVT has also been suggested as a reason for the increased vulnerability of ONHs with an eccentric CRVT [13–15]. In the present study, the location of the CRVT was an independent factor associated with LCD, such that a peripherally located CRVT was associated with a greater LCD. These data support the mechanical hypothesis with respect to the role of the CRVT as a contributor to LC stability in glaucoma. Another finding supporting such a role is that the influence of IOP on LCD was possibly affected by CRVT location; the correlation between LCD and untreated IOP was tend to be stronger in the peripheral CRVT group than in the central CRVT group, although the difference was not statistically significant (*P* = 0.142). Given that IOP is a potent factor with respect to posterior LC displacement [7, 10, 30, 31], it can be speculated that the CRVT supports the LC against IOP-induced displacement.

It is possible that rather than the CRVT itself, the denser connective tissue around the CRVT compared to other parts of the ONH (i.e., superior or inferior pole) could have resisted LC deformation [3, 32]. However, it was not possible to measure the mechanical properties of the LC in this clinical study. The association between LC properties and LC deformation remains to be elucidated.

Meanwhile, it is also possible that the CRVT is pushed toward the periphery as a secondary response LC deformation. Since this was a cross-sectional study, the causal relationship between CRVT location and LCD could not be fully determined. Whether the peripheral location of the CRVT is secondary to LC deformation or a primary factor influencing the susceptibility of the LC to deformation remains to be determined.

In the present study, age was negatively correlated with mean LCD, with younger patients having a deeper LC. This is in line with the findings of previous investigations [33, 34]. Ren et al. showed that LCD was shallower in older eyes than in younger eyes with the same visual-field status and RNFL thickness [33]. Rho et al. also demonstrated a negative correlation between LCD and age after adjusting for IOP and visual-field sensitivity [34]. These findings may be attributable to age-related differences in the properties of the LC tissue. It can be speculated that age-related thickening or stiffening of the LC tissue [7, 35–38] causes the LC to be less reactive to deforming factors (e.g., IOP) in older eyes, while the reverse being true in younger eyes. On the other hand, it was also found herein that the relationship between age and LCD was weaker in the central CRVT group than in the peripheral CRVT group, although the difference was not statistically significant (*P* = 0.070). This may indicate that centrally located CRVTs resist LC deformation even in younger eyes with compliant tissue, which may in part have contributed to the weakened association between age and LCD in this group.

The location of the CRVT was determined in this study based on the anterior LC surface margin, and not on the clinical disc margin. This was because the aim of the study was to determine the effect of CRVT on the LC. In addition, the clinical disc margin is an inconsistent structure, and its determination varies among observers, while the LC margin is a geographically consistent anatomic structure. However, determining the location of the LC insertion at the level of its anterior surface was still difficult even using the images obtained using SS-OCT, which is better able to penetrate the tissue than SD-OCT; the entire LC surface could not be determined in 35.7% (81 of 227 eyes) of the study subjects. However, after adaptive compensation [25], the peripheral anterior LC surface was readily visualized in more than 90% (205 of 227 eyes) of the subjects.

This study was subject to several limitations. First, the study was performed within glaucomatous eyes without control group. Second, the CRVT location was determined based on its exit from the anterior LC surface, although the position of the CRVT in the middle of the LC between the anterior and posterior borders may be more important. However, categorizing the CRVT position in the middle of the LC was not possible because it was usually located within the central one-fifth area of the LC between its anterior and posterior borders. At the level of the posterior LC surface, the CRVT was located at the center of the LC in most of the eyes. The CRVT then ran vertically or obliquely to various degrees through the LC, and at the level of the anterior LC surface, the final CRVT location varied. Third, the center of the radial scans was manually determined by the examiner during OCT, which could have introduced errors in determining the CRVT location. However, because we could not find cases with significant eccentric centering of the radial scans, this factor might have had little effect on the results obtained in this study. Fourth, the LC depth was measured at the deepest points of the LC. When using this method, the depth measurements might have been more distant from the center of the disc in eyes with a central CRVT than in the eyes with a peripheral CRVT, which could have caused LCD to be underestimated in the former eyes. However, measurements were performed in 11 B-scans in the present study, and so most of the images did not contain the CRVT that affects LCD measurements. Fifth, the causal relationship between the CRVT and LCD could not be clarified, as described above. Although a stronger relationship between LCD and untreated IOP or age supports a primary role of CRVT in LC stability, a long-term study on a scale of decades may clarify this issue. Finally, the results of this study may not be generalizable to eyes with tilted or torted optic discs, since such eyes were excluded from the present study in order to eliminate the possible effect of optic-disc tilt or torsion on both CRVT position and LC depth. Moreover, in eyes with severe optic-disc tilt, the peripheral LC was poorly visible and the CRVT was usually hampered by the overlying thick neuroretinal rim.

In conclusion, the position of the CRVT within the LC was associated with the LCD in OAG eyes, together with age, untreated IOP, and global RNFL thickness. Based on the findings of this study, if can be speculated that the CRVT may contribute to the resistance of the LC against deformation. The causal relationship between the position of the CRVT and the LC deformation remains to be determined by a longitudinal study.

## Supporting Information

S1 Table Central retinal vessel trunk dataset. (CSV)
